# Change in D_3_Cr muscle mass in oldest old men and its association with changes in grip strength and walking speed

**DOI:** 10.1371/journal.pone.0320752

**Published:** 2025-04-01

**Authors:** Megan Hetherington-Rauth, Chuck E. McCulloch, William J. Evans, Marc Hellerstein, Mahalakshmi Shankaran, Jane A. Cauley, Kris Ensrud, Lisa Langsetmo, Eric S. Orwoll, Peggy M. Cawthon

**Affiliations:** 1 California Pacific Medical Center, Research Institute, San Francisco, California, United States of America; 2 Department of Epidemiology and Biostatistics, University of California, San Francisco, California, United States of America; 3 Department of Nutritional Sciences and Toxicology, University of California, Berkeley, California, United States of America; 4 Department of Epidemiology, School of Public Health, University of Pittsburgh, Pittsburgh, Pennsylvania, United States of America; 5 Division of Epidemiology and Community Health, University of Minnesota, Minneapolis, Minnesota, United States of America; 6 Oregon Health and Science University, Portland, Oregon, United States of America; Dynamical Business & Science Society - DBSS International SAS, COLOMBIA

## Abstract

**Background:**

The use of lean soft tissue (LST) mass as a surrogate measurement of skeletal muscle mass (SMM) has led to the conclusion that muscle loss is poorly related to functional decline. We hypothesized that when using a more accurate measure of SMM determined by D_3_-creatine dilution (D_3_Cr), longitudinal changes in SMM will be similar in magnitude to changes in strength and physical performance and that skeletal muscle mass will partially mediate the relationship of age with these outcomes.

**Methods:**

We measured change in D_3_Cr muscle mass (kg), handgrip strength (kg), and 6m walk speed (m/s) in 208 men from the Osteoporotic Fractures in Men Study (85.2 ± 4.3 years) over an average of 6.1 years follow-up. Mixed linear effects models adjusted for potential confounders were used to examine the relationship of changes in D_3_Cr muscle mass with changes in grip strength and walking speed.

**Results:**

Annual losses of D_3_Cr muscle mass, grip strength, and walking speed were 2.1%, 2.2%, and 2.6%, respectively (p < 0.001). Each additional kg loss in D_3_Cr muscle mass was associated with a 0.55 kg loss in grip strength and a 0.01 m/s loss in walking speed independent of changes in age (p < 0.001). 41.3% and 22.4% of the relationship between age and loss of grip strength and walking speed, respectively, was attributed to loss of D_3_Cr muscle mass (p < 0.001).

**Conclusion:**

Skeletal muscle mass may have a more important role than previously considered and should not be overlooked as a potentially modifiable determinant in the loss of strength and performance in older age.

## Introduction

Sarcopenia was initially described as the age-associated loss of skeletal muscle mass (SMM) and strength that is associated with functional decline and adverse outcomes including falls, fractures, physical disability and mortality [1]. However, subsequent studies using fat-free mass (all lean components including bone mineral) or lean soft tissue mass (LST) (i.e., all lean components excluding bone mineral) as surrogate measurements of SMM demonstrated a greater age-related decline in strength and function than FFM or LST [2–6]. This lack of relationship resulted in a questioning of the role of SMM in the development of functional decline. Several cross-sectional and longitudinal studies on aging cohorts have suggested that the relationship between SMM as estimated by FFM or LST, functional capacity and health related outcomes to be either weak or negligible, while muscle strength had strong associations with these outcomes [6]. Moreover, a number of studies have indicated that low muscle strength was related to reduced physical function in older adults, irrespective of whether there were concurrent reductions in FFM or LST [6]. These findings have led to alterations in the working definitions of sarcopenia, which now place greater importance on low muscle strength, measured by grip strength, as a diagnostic criterion for sarcopenia [7], and for many to conclude that SMM is relatively unimportant in the declines in muscle function that accompany aging [2,8–10].

However, many of the previous studies have used LST assessed by dual energy x-ray absorptiometry (DXA) to approximate total body or appendicular SMM. The measure of LST includes not only SMM, but non-contractile components, such as fibrotic and connective tissue, organs, and body water [11]. Thus, we posit that the limited association previously reported between “SMM” and adverse health outcomes in older adults is due to the systematic measurement error in the approximation of SMM using DXA LST.

Recently, the use of the d3-creatine dilution (D_3_Cr) method has been highlighted as being a feasible, accurate, and precise measure of total body functional SMM [11]. Low D_3_Cr muscle mass has been found to be associated with poor physical performance [12–14], decreased physical function [3,13], and increased risk of incident fractures [15], falls [12], disability [16,17] and mortality [14,17] in older men and correlated with physical function in older women [18,19], with these relationships not being observed using DXA measures of LST or appendicular LST (ALST). Although these findings suggest that D_3_Cr muscle mass is more highly related to important age‐related health outcomes, there are limited data on changes in D_3_Cr muscle mass. Preliminary data from a sample of 40 men from the Osteoporotic Fractures in Men Study (MrOS) demonstrated that over ~ 1.6 years D_3_Cr muscle mass, grip strength and walking speed declined by about 5% over follow-up, whereas DXA LST did not change [3].

In this study we aimed to build upon these preliminary findings and assess the long-term changes in D_3_Cr muscle mass over ~ 6 years and its association with concurrent changes in grip strength and walking speed in oldest old men from MrOS. Our aims were to 1) describe the relationship between age and changes in D_3_Cr muscle mass, grip strength, and walking speed; 2) evaluate the association of changes in D_3_Cr muscle mass with concurrent changes in grip strength and walking speed; and 3) assess if the association of age with declines in grip strength and walking speed are mediated by age-associated changes in D_3_Cr muscle mass.

## Methods

### Study sample

Community-dwelling ambulatory men aged ≥65 years were enrolled in the multicenter cohort study of aging and osteoporosis (MrOS), which took place across six U.S. clinical sites (Birmingham, AL; Minneapolis, MN; Palo Alto, CA; Monongahela Valley near Pittsburgh, PA; Portland, OR; and San Diego, CA) (https://mrosonline.ucsf.edu/). Recruitment for MrOS took place between April 25, 2000 to March 21, 2002 across all clinical sites. Details of the MrOS study design and recruitment have been published elsewhere [20,21]. All men provided written informed consent, and the study was approved by the Institutional Review Board (IRB) at each center and a centralized Western-Copernicus Group (WCG) IRB and have therefore been performed in accordance with the ethical standards laid down in the 1964 Declaration of Helsinki and its later amendments (IRB approval #20200148). Data for this analysis was accessed on 15/03/2024 and included surviving participants from follow-up Year 14 (2014–2016) and Year 20 (2020–2022) visits who had measures for D_3_Cr muscle mass, physical performance, and strength. At the Year 14 visit, 711 men had complete measures for D_3_Cr muscle mass, grip strength, and walking speed from the 3 clinical sites that were included in the Year 20 visit. Of the 243 men who returned for the Year 20 visit, 208 had complete measures for D_3_Cr muscle mass, grip strength, and walking speed at both time-points. A flow diagram of the men included in the study is depicted in S1 Fig.

### D _3_ Cr muscle mass

The D_3_-creatine (D_3_Cr) dilution method was used to estimate SMM as described previously [12]. In brief, this method involves a participant ingesting a 30-mg dose of stable isotope-labeled creatine (D_3_-creatine), and providing a fasting, morning urine sample 72–144 hours (3–6 days) later in which D_3_-creatinine, unlabeled creatinine, and creatine are measured using high performance liquid chromatography and tandem mass spectroscopy. These measures are then incorporated into an algorithm developed to determine total body creatine pool size and thus SMM [22]. D_3_Cr muscle mass divided by body mass (kg) was calculated to account for variations in total SMM by body size and used in assessing the descriptive characteristics of the sample.

### Physical performance and strength

Grip strength (kg) was assessed by analyzing the maximal value from two tests of each hand using Jamar handheld dynamometers [23]. Walking speed (m/s) was determined by timing the completion of a 6-meter course at the participant’s usual pace and was used as an indicator of physical performance [24].

### Other measures

Participants self-reported physical activity using the Physical Activity Scale for the elderly (PASE) [25] and a physician diagnosis of chronic disease (i.e., diabetes, hyper/hypothyroidism, chronic obstructive pulmonary disease, Parkinson’s disease, dementia/Alzheimer’s disease, congestive heart failure, angina, heart attack, transient ischemic attack, stroke, peripheral vascular disease, high blood pressure, rheumatoid arthritis, osteoarthritis, cancer, and kidney disease) [26]. For analyses, comorbidities were categorized as having 0, 1–2, or > 2 physician diagnoses of chronic disease. The self-reported birthdate at baseline was used to calculate age at the Year 14 and Year 20 visits. Stature was measured by wall-mounted stadiometers and body mass by balance beam or digital scales. Body mass index (BMI) was calculated as body mass (kg)/stature (m^2^). In a subsample of MrOS participants, usual protein intake was estimated by the Block 98.2 MrOS brief FFQ, whose validity and reliability have been previously documented [26].

### Statistical analysis

Baseline characteristics were presented with mean and SD and n (%). Differences between men with and without follow-up data from Year 20 visit were assessed using t-tests for continuous variables and a chi square test for categorical variables.

Mixed effects linear regression models (PROC MIXED procedure in SAS Version 9.1.3, SAS Institute, Cary, NC, USA) were performed to quantify the rate of change in D_3_Cr muscle mass, grip strength and walking speed for each additional year of age. Time was modeled as age at the time of each measurement and centered to the mean age of 86.3 yrs. Models were fit with a random intercept and included age as a fixed effect in the unadjusted model and stature, body mass, physical activity, clinical site, and presence of co-morbidities in the adjusted model. A quadratic term for age was considered to test whether losses in D_3_Cr muscle mass, grip strength, and walking speed increased nonlinearly with increasing age. The Kenward and Roger method was used to estimate degrees of freedom for the tests of fixed effects and generate p-values [27]. Restricted maximal likelihood was used to estimate covariance parameters while assuming an unstructured covariance matrix.

We further used mixed effects linear models to assess if the change in D_3_Cr muscle mass that occurs with age was associated with concurrent changes in grip strength and walking speed. For these models, we separated the within and between individual differences in D_3_Cr muscle mass by including a term for D_3_Cr muscle mass measured at the Year 14 visit to represent between person differences and a term for the change in D_3_Cr muscle mass (i.e., Year 20 visit D_3_Cr muscle mass minus Year 14 visit D_3_Cr muscle mass) to represent longitudinal within person differences. All models were fit with a random intercept and included age in the crude model and the additional covariates of body mass, stature, physical activity, comorbidities, and clinical site as fixed effects in the fully adjusted model.

To assess if the associations between age and changes in grip strength and walking speed were mediated by age-induced changes in D_3_Cr muscle mass, we performed a mediation analysis using a series of mixed linear effects regression models (See S2 Fig for path diagram):

Path 1: Y_ij_ = ß_1_ + *c*X_ij_ + covariates + *γ*_1i_ + *ϵ*_1ij_
Path 2: M_ij_ = ß_2_ + *a*X_ij_ + covariates + *γ*_2i_ + *ϵ*_2ij_
Path 3: Y_ij_ =ß_3_ + *b*M_ij_ + *c’*X_ij_ + covariates + *γ*_3i_ + *ϵ*_3ij_

where Y is either grip strength or walking speed for subject *i* at observation *j*, X is age for subject *i* at observation *j*, M is D_3_Cr muscle mass for subject *i* at observation *j*, *γ* is the random intercept for subject *i*, and *ϵ* is the residual error.

In path 1, *c* describes the total effect (i.e., reflects the sum of the direct effect of age on strength or walking speed and the mediated (indirect) effect of changes in D_3_Cr muscle mass). In path 2, *a* describes the effect of increasing age on D_3_Cr muscle mass. In path 3, *b* describes the unique effect of changes in D_3_Cr muscle mass on strength or walking speed and *c’* represents the direct effect of age on strength or walking speed after accounting for the effect of D_3_Cr muscle mass. The indirect effect is the impact of increasing age on strength or walking speed that is transmitted through changes in D_3_Cr muscle mass and is expressed as *a* x *b*. The proportion mediated was calculated as the ratio between the indirect effect and the total effect. The mediation analysis was performed in R using the *lme4* and *mediation* packages [28,29].

Given the role of protein intake in muscle homeostasis, we evaluated whether adjusting for dietary protein intake affected the results in the subsample of men for whom this was measured. A sensitivity analysis was also performed with only men who had data for both time points.

Model assumptions for the mixed-effects linear models were assessed to ensure validity. Linearity was evaluated using residual plots, normality of residuals and random effects was checked with Q-Q plots, homoscedasticity was assessed by plotting residuals against fitted values, and the random effects structure was validated using likelihood ratio tests and AIC/BIC, with no violations in model assumptions being detected.

## Results

Characteristics of the men at the Year 14 visit from the Palo Alto, Pittsburgh, and Portland clinical sites, who had measures of D_3_Cr muscle mass, grip strength, and walking speed are described in Table 1. The average follow-up time to the Year 20 visit was 6.1 (0.6) years. Men who were able to attend and complete Year 14 and 20 measurements were on average younger, taller, more active, stronger (more grip strength and D_3_Cr muscle mass), walked faster, and had fewer comorbidities compared to men without Year 20 data (p < 0.05).

**Table 1:** Characteristics of the men at the Year 14 visit and comparison of Year 14 visit characteristics between men who were evaluated at Year 20 and those who were not.

	Full sample Year 14 visit (n = 711)	Sample with both follow-up time-points (n = 208)	Sample without Year 20 visit measures (n = 503)	p-value
Age, years	85.2 ± 4.3	83.3 ± 3.1	86.0 ± 4.4	<0.001
Died during follow-up, n(%)			280 (55.7)	
White race, n (%)	632 (88.9)	184 (88.5)	448 (89.1)	0.85
Body mass, kg	79.0 ± 12.2	79.5 ± 10.7	78.7 ± 12.8	0.42
Stature, cm	171.9 ± 6.8	172.9 ± 6.5	171.4 ± 6.9	0.01
BMI, kg/m^2^	26.7 ± 3.6	26.6 ± 3.2	26.8 ± 3.8	0.52
PASE	118.4 ± 63.6	142.8 ± 65.3	108.4 ± 60.1	<0.001
Number of comorbidities, n (%)				0.03
0	100 (14.1)	37 (17.8)	63 (12.5)	
1–2	472 (66.4)	141 (67.8)	331 (65.8)	
3 or more	139 (19.5)	30 (14.4)	109 (21.7)	
D_3_Cr muscle mass, kg	23.9 ± 4.3	25.7 ± 4.0	23.2 ± 4.2	<0.001
D_3_Cr muscle mass/wt, %	31.9 ± 4.9	32.0 ± 5.0	30.1 ± 4.0	0.22
Grip strength, kg	34.8 ± 8.8	38.4 ± 6.8	33.2 ± 9.1	<0.001
6m Walk speed, m/s	1.1 ± 0.3	1.2 ± 0.2	1.0 ± 0.3	<0.001
Dietary protein ^a^, g/d	61.6 ± 24.5	59.1 ± 22.3	62.7 ± 25.4	0.15

*Note.* PASE, Physical Activity Scale for the Elderly^22^; D_3_Cr, D_3_-creatine dilution

^a^Full sample Year 14 n = 425; Sample with both follow-up time-points n = 132; Sample without Year 20 n = 293

**Fig 1:**
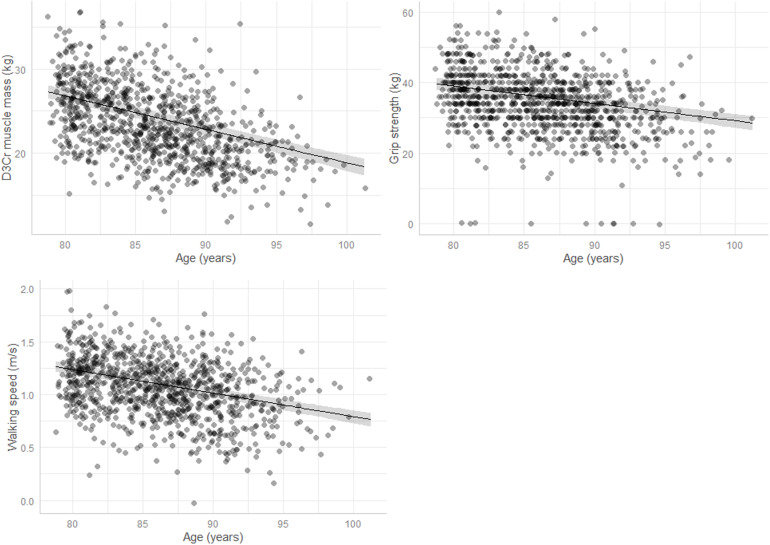
Relationship between increasing age and changes in a) D3Cr muscle mass, b) grip strength, and c) walking speed.

As depicted in Fig 1, estimated mean D_3_Cr muscle mass, grip strength, and walking speed decreased with increasing age, with an estimated annual change in D_3_Cr muscle mass of −0.53 kg/yr (95% CI: −0.58, −0.48), change in grip strength of −0.78 kg/yr (95% CI: −0.87, −0.69), and change in walking speed of −0.03 m/s/yr (95% CI: −0.03, −0.02). These associations were slightly attenuated after adjusting for body mass, stature, physical activity, comorbidities, and clinical site (−0.40 kg D_3_Cr muscle mass, 95% CI: −0.45, −0.35; −0.49 kg grip strength, 95% CI: −0.59, −0.40; −0.02 m/s walking speed, 95% CI: −0.03, −0.02). There was no indication of a curvilinear relationship of age with D_3_Cr muscle mass, strength, and performance measures (β-coefficient for the age^2^ (quadratic) term p > 0.05). Table 2 shows the estimated change in D_3_Cr muscle mass, grip strength, and walking speed between follow-up Year 14 and Year 20 visits derived from the linear mixed effects models. The greatest percent changes were seen for walking speed (−15%), followed by similar declines in grip strength and D_3_Cr muscle mass, which declined by a similar magnitude (−13%) over the 6-year timeframe. Estimated changes were similar after further adjusting for dietary protein intake (S1 Table).

**Table 2:** Estimated change in D_3_Cr muscle mass, grip strength, and walking speed between follow-up Year 14 and Year 20 visits (Average Follow-Up, 6.1 yrs) for oldest old men [Mean (SD].

	Absolute change	Percent change	Annualized change	Annualized percent change
D_3_Cr muscle mass (kg)				
Unadjusted^a^	−3.25 (0.31)	−13.19 (1.57)	−0.53 (0.00)	−2.17 (0.16)
Adjusted^b^	−3.18 (1.02)	−12.86 (4.07)	−0.52 (0.16)	−2.12 (0.66)
Grip strength (kg)				
Unadjusted^a^	−4.74 (0.45)	−13.23 (1.58)	−0.78 (0.00)	−2.18 (0.16)
Adjusted^b^	−4.81 (1.96)	−13.21 (5.20)	−0.79 (0.32)	−2.17 (0.85)
Walking speed (m/s)				
Unadjusted^a^	−0.17 (0.02)	−15.25 (1.93)	−0.03 (0.00)	−2.51 (0.22)
Adjusted^b^	−0.18 (0.07)	−15.70 (5.83)	−0.03 (0.01)	−2.59 (0.97)

*Note.* D_3_Cr, D_3_-creatine dilution.

^a^Change estimated using linear mixed effects models.

^b^Adjusted for body mass, stature, physical activity, comorbidities, and clinical site

Associations between longitudinal changes in D_3_Cr muscle mass and changes in grip strength and walking speed are reported in Table 3. Each additional kg of within-person change in D_3_Cr muscle mass was associated with a 0.44 kg change in grip strength and a 0.01 m/s change in walking speed independent of changes in age and additional confounders (i.e., body mass, stature, physical activity, comorbidities, and clinical site). Hence, a man with an average baseline grip strength and walking speed who lost 5 kg D_3_Cr muscle mass over 6 years would be expected to have a grip strength of 32.6 kg and walking speed of 1.01 m/s, whereas a man who lost 1 kg D_3_Cr muscle mass with a similar Year 14 D_3_Cr muscle mass, age and other characteristics (i.e., body mass, stature, physical activity level, and medical conditions) would be expected to have a grip strength of 34.3 kg and walking speed of 1.06 m/s. Year 14 between-person differences in D_3_Cr muscle mass were related to changes in both grip strength and walking speed (p < 0.05). Further adjustment for dietary protein intake did not alter associations (S2 Table).

**Table 3:** Association of longitudinal changes in D_3_Cr muscle mass with concurrent changes in grip strength and walking speed.

	Minimally adjusted^a^		Multivariable adjusted^b^	
	β (95% CI)	p-value	β (95% CI)	p-value
Grip Strength (kg)				
Year 14 Visit D_3_Cr muscle mass (between-person difference)	0.78 (0.64, 0.92)	<0.0001	0.56 (0.41, 0.71)	<0.0001
6-year change in D_3_Cr muscle mass (within-person change)	0.55 (0.39, 0.71)	<0.0001	0.44 (0.28, 0.60)	<0.0001
Walking speed (m/s)				
Year 14 Visit D_3_Cr muscle mass (between-person difference)	0.011 (0.007, 0.016)	<0.0001	0.013 (0.008, 0.017)	<0.0001
6-year change in D_3_Cr muscle mass (within-person change)	0.013 (0.007, 0.019)	<0.0001	0.011 (0.006, 0.017)	<0.0001

*Note.* D_3_Cr, D_3_-creatine dilution.

^a^Adjusted for age.

^b^Adjusted for age, body mass, stature, physical activity, comorbidities, and clinical site

Given the observed relationship between age and D_3_Cr muscle mass, grip strength, and walking speed and the relationship of D_3_Cr muscle mass with grip strength and walking speed, we further assessed if the age relationship with grip strength and walking speed was mediated by age-induced changes on D_3_Cr muscle mass. Results of the mediation analysis are presented in Table 4. We observed a significant indirect effect of age on grip strength through D_3_Cr muscle mass, such that 42% of the relationship between age and loss of grip strength can be attributed to loss of D_3_Cr muscle mass. Similarly, but to a lesser magnitude, D_3_Cr muscle mass mediated 22% of the relationship between age and slowing of walking speed. Hence, declines in grip strength and walking speed with age are partially due to the age-related decline of D_3_Cr muscle mass. The percentage mediated did not substantially change after adjusting for dietary protein intake, although the value was slightly reduced for walking speed (18.2%) (S3 Table).

**Table 4:** Mediation of D_3_Cr muscle mass on age and grip strength and walking speed relationship.

	Total effect of Age on outcome (Path 1, β = *c*)		Effect of Age on D_3_Cr muscle mass (Path 2, β = ^a^)		Unique effect of D_3_Cr muscle mass on outcome (Path 3, β = *b*)		Direct effect of Age on outcome (Path 3, β = *c’*)		Indirect effect (β = a * *b*)		Proportion Mediated (%)
	β (95% CI)	p−value	β (95% CI)	p−value	β (95% CI)	p−value	β (95% CI)	p−value	β (95% CI)	p−value	
Grip strength, kg	−0.49 (−0.59, −0.38)	<0.0001	−0.40 (−0.45, −0.35)	<0.0001	0.51 (0.38, 0.64)	<0.0001	−0.28 (−0.40, −0.17)	<0.0001	−0.20 (−0.26, −0.15)	<0.0001	41.3 (29.0, 58.0)
Walking speed, m/s	−0.022 (−0.025, −0.020)	<0.0001	−0.40 (−0.45, −0.35)	<0.0001	0.012 (0.008, 0.016)	<0.0001	−0.017 (−0.021, −0.014)	<0.0001	−0.005 (−0.007, −0.000)	<0.0001	22.4 (13.9, 31.0)

*Note.* D_3_Cr, D_3_−creatine dilution

^a^All models adjusted for body mass, stature, physical activity, comorbidities, and clinical site

When including only men with complete data for both Year 14 and Year 20 visits (n = 208), we observed similar results for the estimated change in D_3_Cr muscle mass, grip strength, and walking speed with and without adjustment for body mass, stature, physical activity, comorbidities, and clinical site (S4 Table). Associations between longitudinal changes in D_3_Cr muscle mass and changes in grip strength and walking speed were slightly weaker when removing men with missing data and were no longer significant for walking speed (S5 Table). Results from mediation analyses still showed that the D_3_Cr muscle mass mediated the relationship of age with grip strength and walking speed, however the percent mediated was slightly greater for grip strength (45.4%) and less for walking speed (14.5%) (S6 Table).

## Discussion

Our findings suggest that loss of SMM as measured using the D_3_Cr method is an important contributor to loss of muscle strength and performance in oldest old men. In oldest old men, D_3_Cr muscle mass, grip strength, and walking speed continued to decrease with age. Changes in D_3_Cr muscle mass were associated with changes in grip strength and walking speed, such that the effect of age on grip strength and walking speed was partially explained by age−related losses in D_3_Cr muscle mass.

There has been debate in the field as to the importance of SMM as a key characteristic in defining sarcopenia. While there is no disagreement among experts that SMM is reduced with advancing age, the degree to which this reduction is associated with loss of strength and performance has been questioned. The debate has arisen from observations of a dissociation between loss of surrogate measures of SMM (i.e., LST, muscle cross−sectional area (CSA), and FFM) with loss of strength, with much steeper declines in strength being observed compared to these surrogate measures with advancing age. For instance, annual losses in LST, muscle CSA, and FFM for men ≥70 years have been reported to be 0.80%, 0.98%, and 0.18%, respectively, with even steeper declines of 3−4% reported for muscle strength [30]. Moreover, despite increases in leg LST and CSA in men who gained body mass during 5−years follow−up, these gains did not prevent age−related loss of leg muscle isometric strength [2,31]. Our results, on the other hand, indicated that loses of SMM as assessed with D_3_Cr dilution were related to and tracked linearly with losses in strength, with annual loses of ~ 2% among oldest old men.

The relationship between SMM and performance has also been inconsistent in the literature. In a meta−analysis of cross−sectional studies of older adults, low “SMM” estimated either by DXA (i.e., LST or ALST), bioelectrical impedance (BIA) (i.e., FFM), or computed tomography (CT) CSA of a single muscle group was found to be associated with poor physical performance for only 35% of the functional measures of disability tested [32]. When focusing on solely longitudinal studies, a meta−analysis assessing the relationship of LST, muscle CSA, and FFM with measures of functional decline (mainly self−reported activities of daily living and/or walking speed) concluded that low SMM was not associated with the loss of physical performance with age, despite the fact that SMM was not in fact measured [10]. Contrarily, we observed that changes in SMM with age were associated with concurrent changes in walking speed in oldest old men, albeit modest in magnitude.

It is likely that part of the inconsistent results across previous studies can be attributed to the method used to estimate SMM. With aging, the intercellular gap between muscle fibers increases due to the infiltration of intramuscular adipose tissue and non−contractile fibrotic tissue [33], and the extracellular water relative to intracellular increases due to decreases in cell volume [34]. Previous studies have mainly relied on DXA, BIA, or CT to estimate SMM, however, none of these methods directly measure SMM [35]. For instance, DXA measures LST and not muscle, with intramuscular fat, connective and fibrotic tissue, water, and organ mass included as part of LST [11]. When comparing DXA and D_3_Cr in a sample of older men from the MrOS population at the Year 14 visit (n = 1,376), Orwoll et al. found that D_3_Cr muscle mass was only about 50% of LST [36]. Similarly, Cawthon et al. observed only a moderate correlation between D_3_Cr muscle mass and DXA LST, with a substantial overestimation of SMM by DXA and no significant relationship between percent SMM and ALST/ht [12]. BIA also does not measure SMM. It measures the impedance of various tissues based on their water and ion concentration to an electrical current and incorporates this information into population−specific prediction equations, which are commonly validated against DXA, to obtain an estimate of FFM or LST [37]. Thus, BIA is highly sensitive to hydration status and electrolyte balance, factors known to vary in older adults, as well as to the equation used to predict FFM or LST [37]. CT usually measures the volume of individual muscle groups rather than total body SMM, which can alter results, given muscle−specific differences in the response to aging [38]. The D_3_Cr dilution method measures the total body creatine pool size and, thus skeletal muscle contractile mass [39], the cellular site of muscle force−generating capacity [33]. When using this more accurate way to assess whole body SMM that can capture and account for age related changes occurring in muscle tissue, we found changes in SMM to be an important component of age−related loss of muscle strength and performance independent of changes in physical activity levels and age−associated chronic diseases. Other commonly used proxy measures of SMM (e.g., FFM or LST) were not assessed at the Year 20 follow−up visit, thus, although not comparable by nature as these other measures represent different constructs, we were unable to see how age−related changes in these measures differed from that of D_3_Cr muscle mass. Nevertheless, the current findings extend previous cross−sectional findings demonstrating the association of decreased D_3_Cr muscle mass with lower muscle strength and walking speed [13,18,36].

Although changes in SMM with age are related to the declines in the force−generating capacity of muscle, cellular and molecular factors at the single muscle fiber level and at the level of the neuromuscular system are also altered with age, and contribute to the loss of muscle strength and performance independent of changes in muscle size/mass. Such factors include weaker excitation−contraction coupling due to decreased calcium sensitivity and release, mitochondrial dysfunction and diminished respiratory capacity, lower myosin concentrations resulting in reduced force generation, decrease in the proportion of Type II muscle fibers, declines in motor unit recruitment, and reductions in corticospinal excitability [40–42]. Despite all these SMM independent factors, we still found that ~ 41% of the relationship between increasing age with changes in grip strength was mediated by age−related declines in D_3_Cr muscle mass. We also observed that age−related declines in D_3_Cr muscle mass were partially responsible for the slowing of walking speed with age, although to a slightly lesser degree than muscle strength (~22%). This is likely due to walking being a more complex action affected by additional SMM independent factors, such as age−induced changes in sensorimotor and cognitive processes involved in balance, attention, and motivation [43]. Habitual walking speed has been found to be strongly associated with maximal aerobic capacity (VO_2max_) in older men and women [44]. Previous data from the Baltimore Longitudinal Study on Aging demonstrated that a substantial component of the age−related decrease in VO_2max_ resulted from declining SMM [45]. Overall, deciphering the predominant driving mechanisms for the loss of strength and performance with advancing age is challenging, as they are multifactorial in nature. Nevertheless, these results highlight the classic biological concept of “function follows form”, with physiological functions being mechanistically linked to their structural/anatomic source [46]. Therefore, SMM should not be overlooked as an important modifiable determinant in the loss of strength and performance in older age.

It is important to note that this study in not without limitations. As with any longitudinal study on aging, there will be survivor bias. For instance, men who survived but did not attend the follow−up visit most likely had greater declines in SMM, grip strength, and walking speed. We did not observe major differences in results when excluding men with missing data from the analyses, although the relationship between changes in D_3_Cr muscle mass and changes in walking speed was no longer significant, perhaps reflecting leg power. This study also included only older men, restricting the generalization of our findings to women, which is of importance considering the well−established sex differences in body composition [47]. In addition, the MrOS cohort was composed of relatively healthy men representing a narrow age range (i.e., early 80s to early 90s), thus declines in SMM, strength, and performance may be lower and decline at different rates than that of the general population of that age. Although we did not observe non−linear declines in D_3_Cr muscle mass, strength, and performance with age, it is possible that greater rates of decline with increasing age could have been observed if we had a cohort with a larger age range. Lastly, like all body composition assessment techniques D_3_Cr dilution relies on underlying assumptions that may not always be met, leading to inherent errors and limitations in its accuracy and applicability [48]. First, a small variable amount of labeled D_3_Cr is “spilled” into the urine, and although a correction factor has been incorporated into the estimation of D_3_Cr muscle mass, it was validated for ages 20−81 years [22], and thus may not be fully valid for oldest−old adults, like in our study. Second, the concentration of creatine is assumed to be 4.3 g/kg wet weight of muscle, however, this number has been shown to vary between 3.8 to 5.4 g/kg depending on muscle fiber composition, age, and physical activity level, although these large deviations are mostly seen in young males, athletic populations, and certain wasting disease states [48,49]. Diet can also influence creatine concentration; however, dietary creatine intake is minimal and contributes only a small fraction to the total creatine pool, with significant increases in muscle creatine content only occurring with high−dose supplementation (>5g/d) [50]. We did not have data on supplemental creatine use among the MrOS participants; however, it is unlikely that most of the men were consuming high doses of creatine supplements.

Despite limitations, this is one of the few longitudinal studies to assess the relationship of D_3_Cr muscle mass with the major sarcopenic outcomes of grip strength and walking speed in an older adult population. Using a subsample from the same MrOS cohort who had repeat measures for both DXA LM and D_3_Cr muscle mass, Duchowny et al. observed weak to moderate correlations between changes in D_3_Cr muscle mass with changes in grip strength and walking speed, respectively, however these correlations did not reach statistical significance [3]. These results, differed from our significant findings of changes in D_3_Cr muscle mass with concurrent changes in grip strength and walking speed. It is likely that differences in results stem from lack of adequate power to detect significant associations (i.e., small sample size of n = 40) and short follow−up time (1.6 years) in the Duchowny et al. pilot study [3]. Moreover, the analytical approach of using simple correlations between change in D_3_Cr muscle mass and change in the outcomes does not consider the great variability between individuals over time. We were able to account for this with our use of mixed effects regression (an approach not possible in smaller samples), which allows for a random intercept. The random intercept accounts for the fact that each individual may start with a different baseline level of D_3_Cr muscle mass, strength, and/or function. This allows the model to account for these baseline differences when assessing the relationship between changes in D_3_Cr muscle mass and changes in strength/function over time. Nevertheless, future longitudinal studies that include women and with a large age range, as well as important confounders (e.g., objective PA assessment, supplementary Cr use) are needed to confirm our findings.

## Conclusion

Men in the last decades of life experience previously unreported losses in SMM, strength, and habitual walking speed with similar annual losses of ~ 2% for D_3_Cr muscle mass and grip strength and ~ 3% for walking speed. Our findings suggest that SMM itself plays a more important role in the loss of strength and performance with age than has been previously considered. Future studies should focus on longitudinal associations of D_3_Cr muscle mass with not only strength and physical performance, but also functional limitations and disability, which could provide more insight into the clinical utility of D_3_Cr muscle mass as a diagnostic criterion for sarcopenia.

## Supporting information

S1 Fig Flow diagram of study sample. (TIFF)

S2 Fig Mediation path diagram. (PDF)

S1 Table Estimated change in D_3_Cr muscle mass, grip strength, and walking speed between follow−up Year 14 and Year 20 visits (Average Follow−Up, 6.1 yrs) for oldest old men after adjusting for dietary protein intake (g/d) (n = 434) [Mean (SD]. (DOCX)

S2 Table Association of longitudinal changes in D_3_Cr muscle mass with concurrent changes in grip strength and walking speed after adjusting for dietary protein intake (n = 434). (DOCX)

S3 Table Mediation of D_3_Cr muscle mass on age and grip strength and walking speed relationship adjusted for dietary protein intake (DOCX)

S4 Table Estimated change in D_3_Cr muscle mass, grip strength, and walking speed between follow−up Year 14 and Year 20 visits (Average Follow−Up, 6.1 yrs) for oldest old men with complete measures for both time points (n = 208) [Mean (SD] (DOCX)

S5 Table Association of longitudinal changes in D_3_Cr muscle mass with concurrent changes in grip strength and walking speed in men with complete measures for the Year 14 and Year 20 visits (n = 208). (DOCX)

S6 Table Mediation of D3Cr muscle mass on age and grip strength and walking speed relationship in men with complete measures for the Year 14 and Year 20 visits (n = 208) (DOCX)

S1 STROBE checklist Key reporting items for observational studies. (DOCX)
