# Supplementary figures and images for: Change in D3Cr muscle mass in oldest old men and its association with changes in grip strength and walking speed
Source: PLoS One. 2025 Apr 1;20(4):e0320752. doi: 10.1371/journal.pone.0320752 (PMC11960989; doi:10.1371/journal.pone.0320752)

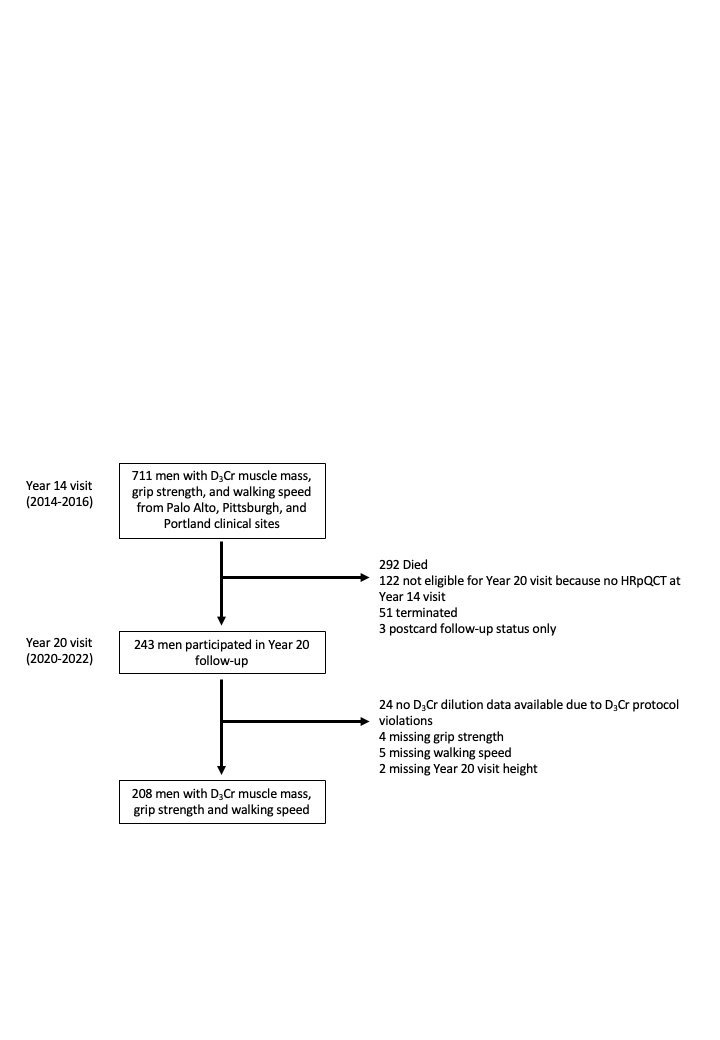

Supplement: S1 Fig — (TIFF) [file pone.0320752.s001.tiff]
