# Supplementary material for: Change in D3Cr muscle mass in oldest old men and its association with changes in grip strength and walking speed
Source: PLoS One. 2025 Apr 1;20(4):e0320752. doi: 10.1371/journal.pone.0320752 (PMC11960989; doi:10.1371/journal.pone.0320752)
Supplement: S2 Fig — (PDF) [file pone.0320752.s002.pdf]

S2 Fig. Mediation path diagram

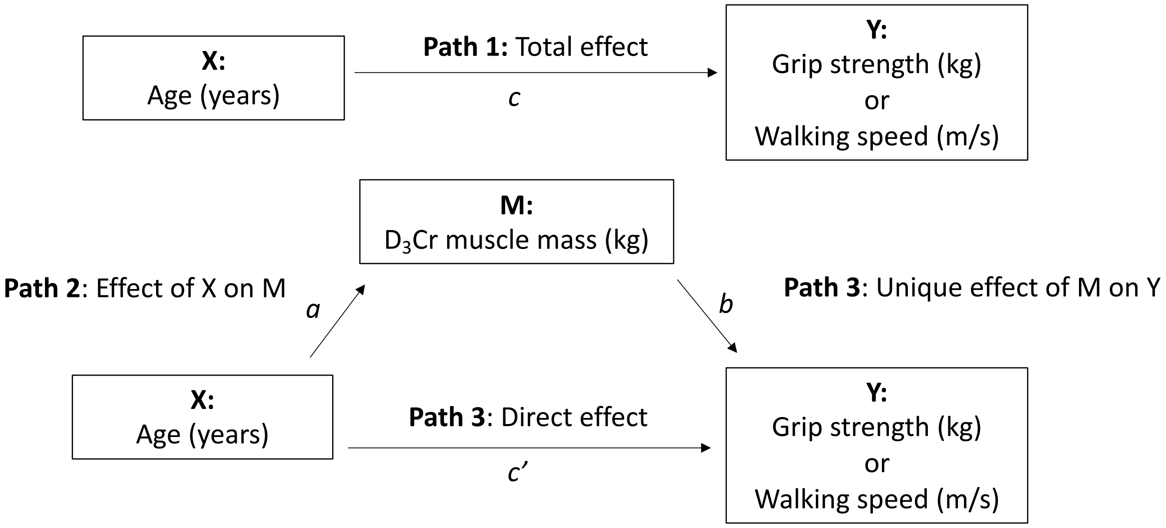

Path 1: $Y_{ij}=ß_1 + cX_{ij} + \text{covariates} + \gamma_{1i} + \epsilon_{1ij}$

Path 2: $M_{ij}= ß_2 + aX_{ij} + \text{covariates} + \gamma_{2i} + \epsilon_{2ij}$

Path 3: $Y_{ij}=ß_3 + bM_{ij} + c'X_{ij} + \text{covariates} + \gamma_{3i} + \epsilon_{3ij}$

where Y is either grip strength or walking speed for subject *i* at observation *j*, X is age for subject *i* at observation *j*, M is D_3Cr muscle mass for subject *i* at observation *j*, γ is the random intercept for subject *i*, and ϵ is the residual error.

In path 1, *c* describes the total effect (i.e., reflects the sum of the direct effect of age on strength or walking speed and the mediated (indirect) effect of changes in D_3Cr muscle mass). In path 2, *a* describes the effect of increasing age on D_3Cr muscle mass. In path 3, *b* describes the unique effect of changes in D_3Cr muscle mass on strength or walking speed and *c′* represents the direct effect of age on strength or walking speed after accounting for the effect of D_3Cr muscle mass. The indirect effect is the impact of increasing age on strength or walking speed that is

transmitted through changes in D_3Cr muscle mass and is expressed as *a* x *b*. The proportion mediated was calculated as the ratio between the indirect effect and the total effect.
