# Supplementary material for: Change in D3Cr muscle mass in oldest old men and its association with changes in grip strength and walking speed
Source: PLoS One. 2025 Apr 1;20(4):e0320752. doi: 10.1371/journal.pone.0320752 (PMC11960989; doi:10.1371/journal.pone.0320752)
Supplement: S1 Table — (DOCX) [file pone.0320752.s003.docx]

**S1 Table.** Estimated change in D_3_Cr muscle mass, grip strength, and walking speed between follow-up Year 14 and Year 20 visits (Average Follow-Up, 6.1 yrs) for oldest old men after adjusting for dietary protein intake (g/d) (n=434) [Mean (SD].

|  | Absolute change | Percent change | Annualized change | Annualized percent change |
| --- | --- | --- | --- | --- |
|  |  |  |  |  |
| D_3_Cr muscle mass (kg) | -3.24 (0.98) | -13.1 (4.0) | -0.55 (0.16) | -2.24 (0.67) |
|  |  |  |  |  |
|  |  |  |  |  |
| Grip strength (kg) | -4.72 (1.77) | -12.95 (4.64) | -0.81 (0.30) | -2.21 (0.79) |
|  |  |  |  |  |
|  |  |  |  |  |
| Walking speed (m/s) | -0.17 (0.06) | -14.85 (4.89) | -0.03 (0.01) | -2.54 (0.84) |
| *Note.* D_3_Cr, D_3_-creatine dilution  ^a^ Change estimated using linear mixed effects models  ^b^ All models adjusted for body mass, stature, physical activity, comorbidities, clinical site, and protein intake | | | | |
