# Supplementary material for: Change in D3Cr muscle mass in oldest old men and its association with changes in grip strength and walking speed
Source: PLoS One. 2025 Apr 1;20(4):e0320752. doi: 10.1371/journal.pone.0320752 (PMC11960989; doi:10.1371/journal.pone.0320752)
Supplement: S2 Table — (DOCX) [file pone.0320752.s004.docx]

**S2 Table.** Association of longitudinal changes in D_3_Cr muscle mass with concurrent changes in grip strength and walking speed after adjusting for dietary protein intake (n=434).

|  | Multivariable adjusted^a^ | |
| --- | --- | --- |
|  | β (95% CI) | p-value |
| Grip Strength (kg) |  |  |
| Year 14 Visit D_3_Cr muscle mass  (between-person difference) | 0.55 (0.34, 0.75) | <0.0001 |
| 6-year change in D_3_Cr muscle mass  (within-person change) | 0.46 (0.25, 0.67) | <0.0001 |
|  |  |  |
| Walking speed (m/s) |  |  |
| Year 14 Visit D_3_Cr muscle mass  (between-person difference) | 0.011 (0.005, 0.017) | 0.0003 |
| 6-year change in D_3_Cr muscle mass  (within-person change) | 0.008 (0.001, 0.015) | 0.03 |
| *Note.* D_3_Cr, D_3_-creatine dilution  ^a^Adjusted for age, body mass, stature, physical activity, comorbidities, clinical site and protein intake | | |
