# Supplementary material for: Change in D3Cr muscle mass in oldest old men and its association with changes in grip strength and walking speed
Source: PLoS One. 2025 Apr 1;20(4):e0320752. doi: 10.1371/journal.pone.0320752 (PMC11960989; doi:10.1371/journal.pone.0320752)
Supplement: S3 Table — (DOCX) [file pone.0320752.s005.docx]

**S3 Table.** Mediation of D_3_Cr muscle mass on age and grip strength and walking speed relationship adjusted for dietary protein intake

|  | **Total effect of Age on outcome**  **(Path 1, β = *c*)** | | **Effect of Age on D_3_Cr muscle mass**  **(Path 2, β = *a*)** | | **Unique effect of D_3_Cr muscle mass on outcome**  **(Path 3, β = *b*)** | | **Direct effect of Age on outcome**  **(Path 3, β = *c'*)** | | **Indirect effect**  **(β = *a***b*)** | | **Proportion Mediated (%)** |
| --- | --- | --- | --- | --- | --- | --- | --- | --- | --- | --- | --- |
|  | β (95% CI) | p-value | β (95% CI) | p-value | β (95% CI) | p-value | β (95% CI) | p-value | β (95% CI) | p-value |  |
| Grip strength, kg | -0.51 (-0.64, -0.37) | 0.0001 | -0.42 (-0.49, -0.35) | <0.0001 | 0.50 (0.33, 0.67) | <0.0001 | -0.31 (-0.47, -0.14) | <0.0001 | -0.22 (-0.30, -0.14) | <0.0001 | 41.6 (25.5, 64.0) |
| Walking speed, m/s | -0.022 (-0.027, -0.018) | <0.0001 | -0.42 (-0.49, -0.35) | <0.0001 | 0.009 (0.004, 0.015) | 0.0005 | -0.018 (-0.023, -0.014) | <0.0001 | -0.004 (-0.007, -0.000) | <0.0001 | 18.2 (7.3, 30.0) |
| *Note.* D_3_Cr, D_3_-creatine dilution  ^a^ All models adjusted for body mass, stature, physical activity, comorbidities, clinical site, and protein intake | | | | | | | | | | | |
