# Supplementary material for: Change in D3Cr muscle mass in oldest old men and its association with changes in grip strength and walking speed
Source: PLoS One. 2025 Apr 1;20(4):e0320752. doi: 10.1371/journal.pone.0320752 (PMC11960989; doi:10.1371/journal.pone.0320752)
Supplement: S4 Table — (DOCX) [file pone.0320752.s006.docx]

**S4 Table.** Estimated change in D_3_Cr muscle mass, grip strength, and walking speed between follow-up Year 14 and Year 20 visits (Average Follow-Up, 6.1 yrs) for oldest old men with complete measures for both time points (n=208) [Mean (SD].

|  | Absolute change | Percent change | Annualized change | Annualized percent change |
| --- | --- | --- | --- | --- |
| D_3_Cr muscle mass (kg) |  |  |  |  |
| Unadjusted^a^ | -3.48 (0.34) | -13.75 (1.72) | -0.57 (0.00) | -2.26 (0.17) |
| Adjusted^b^ | -3.62 (0.97) | -14.25 (3.94) | -0.59 (0.15) | -2.35 (0.64) |
|  |  |  |  |  |
| Grip strength (kg) |  |  |  |  |
| Unadjusted^a^ | -4.67 (0.46) | -12.29 (1.48) | -0.77 (0.00) | -2.02 (0.13) |
| Adjusted^b^ | -4.77 (1.64) | -12.53 (4.22) | -0.79 (0.27) | -2.07 (0.69) |
|  |  |  |  |  |
| Walking speed (m/s) |  |  |  |  |
| Unadjusted^a^ | -0.20 (0.02) | -16.68 (2.28) | -0.03 (0.00) | -2.74 (0.25) |
| Adjusted^b^ | -0.20 (0.04) | -16.63 (3.70) | -0.03 (0.01) | -2.74 (0.58) |
| *Note.* D_3_Cr, D_3_-creatine dilution  ^a^Change estimated using linear mixed effects models  ^b^Adjusted for body mass, stature, physical activity, comorbidities, and clinical site | | | | |
