# Supplementary material for: Change in D3Cr muscle mass in oldest old men and its association with changes in grip strength and walking speed
Source: PLoS One. 2025 Apr 1;20(4):e0320752. doi: 10.1371/journal.pone.0320752 (PMC11960989; doi:10.1371/journal.pone.0320752)
Supplement: S5 Table — (DOCX) [file pone.0320752.s007.docx]

**S5 Table.** Association of longitudinal changes in D_3_Cr muscle mass with concurrent changes in grip strength and walking speed in men with complete measures for the Year 14 and Year 20 visits (n=208).

|  | Minimally adjusted^a^ | |  | Multivariable adjusted^b^ | |
| --- | --- | --- | --- | --- | --- |
|  | β (95% CI) | p-value |  | β (95% CI) | p-value |
| Grip Strength (kg) |  |  |  |  |  |
| Year 14 Visit D_3_Cr muscle mass  (between-person difference) | 0.60 (0.38, 0.81) | <0.0001 |  | 0.44 (0.21, 0.66) | <0.0001 |
| 6-year change in D_3_Cr muscle mass  (within-person change) | 0.44 (0.25, 0.63) | <0.0001 |  | 0.36 (0.17, 0.55) | <0.0003 |
|  |  |  |  |  |  |
| Walking speed (m/s) |  |  |  |  |  |
| Year 14 Visit D_3_Cr muscle mass  (between-person difference) | 0.004 (-0.003, 0.011) | 0.280 |  | 0.004 (-0.003, 0.011) | 0.241 |
| 6-year change in D_3_Cr muscle mass  (within-person change) | 0.006 (-0.0003, 0.013) | 0.060 |  | 0.005 (-0.001, 0.012) | 0.125 |
| *Note.* D_3_Cr, D_3_-creatine dilution  ^a^Adjusted for age  ^b^Adjusted for age, body mass, stature, physical activity, comorbidities, and clinical site | | | | | |
