# Supplementary material for: Change in D3Cr muscle mass in oldest old men and its association with changes in grip strength and walking speed
Source: PLoS One. 2025 Apr 1;20(4):e0320752. doi: 10.1371/journal.pone.0320752 (PMC11960989; doi:10.1371/journal.pone.0320752)
Supplement: S6 Table — (DOCX) [file pone.0320752.s008.docx]

**S6 Table.** Mediation of D_3_Cr muscle mass on age and grip strength and walking speed relationship in men with complete measures for the Year 14 and Year 20 visits (n=208).

|  | **Total effect of Age on outcome**  **(Path 1, β = *c*)** | | **Effect of Age on D_3_Cr muscle mass**  **(Path 2, β = *a*)** | | **Unique effect of D_3_Cr muscle mass on outcome**  **(Path 3, β = *b*)** | | **Direct effect of Age on outcome**  **(Path 3, β = *c'*)** | | **Indirect effect**  **(β = *a***b*)** | | **Proportion Mediated (%)** |
| --- | --- | --- | --- | --- | --- | --- | --- | --- | --- | --- | --- |
|  | β (95% CI) | p-value | β (95% CI) | p-value | β (95% CI) | p-value | β (95% CI) | p-value | β (95% CI) | p-value |  |
| Grip strength, kg | -0.54 (-0.67, -0.40) | <0.0001 | -0.50 (-0.58, -0.43) | <0.0001 | 0.49 (0.33, 0.65) | <0.0001 | -0.29 (-0.44, -0.13) | <0.0001 | -0.25 (-0.34, -0.16) | <0.0001 | 45.3 (29.2, 69.0) |
| Walking speed, m/s | -0.029 (-0.033, -0.020) | <0.0001 | -0.50 (-0.58, -0.43) | <0.0001 | 0.008 (0.003, 0.014) | 0.002 | -0.024 (-0.029, -0.020) | <0.0001 | -0.004 (-0.007, -0.000) | 0.002 | 14.5 (4.61, 25.0) |

*Note.* D_3_Cr, D_3_-creatine dilution

^a^Adjusted for body mass, stature physical activity, comorbidities, and clinical site
